# Exposure to Bullying or Hazing During Deployment and Mental Health Outcomes Among US Army Soldiers

**DOI:** 10.1001/jamanetworkopen.2022.52109

**Published:** 2023-01-24

**Authors:** Laura Campbell-Sills, Xiaoying Sun, Ronald C. Kessler, Robert J. Ursano, Sonia Jain, Murray B. Stein

**Affiliations:** 1 Department of Psychiatry, University of California, San Diego, La Jolla; 2 Herbert Wertheim School of Public Health and Human Longevity Science, University of California, San Diego, La Jolla; 3 Department of Health Care Policy, Harvard Medical School, Boston, Massachusetts; 4 Center for the Study of Traumatic Stress, Department of Psychiatry, Uniformed Services University of the Health Sciences, Bethesda, Maryland; 5 Veterans Affairs San Diego Healthcare System, San Diego, California

## Abstract

**Importance:**

Workplace bullying is associated with mental disorders and suicidality in civilians, but few studies have examined associations of bullying with these outcomes among military personnel.

**Objective:**

To evaluate associations of being bullied or hazed during deployment with major depressive disorder (MDD), intermittent explosive disorder, posttraumatic stress disorder (PTSD), suicidal ideation, and substance use disorder (SUD).

**Design, Setting, and Participants:**

This cohort study used data from the Army Study to Assess Risk and Resilience in Servicemembers (Army STARRS) New Soldier Study (NSS; April 1, 2011, to November 30, 2012) and wave 1 of the STARRS Longitudinal Study (STARRS-LS1; September 1, 2016, to April 30, 2018). A computerized survey administered at 3 US Army installations (NSS) and a web/telephone survey (STARRS-LS1) were used to collect data. Data were analyzed from October 11, 2021, to October 28, 2022. The STARRS-LS1 recruited a probability sample of active-duty soldiers and veterans who had participated in Army STARRS baseline surveys while on active duty (weighted response rate, 35.6%). Respondents whose baseline was the NSS and who had deployed to a combat theater at least once were eligible for this study.

**Exposures:**

Being bullied or hazed during a combat deployment.

**Main Outcomes and Measures:**

The primary outcomes were MDD, intermittent explosive disorder, PTSD, and suicidal ideation in the 12 months before STARRS-LS1 and SUD in the 30 days before STARRS-LS1, assessed with items from the Composite International Diagnostic Interview Screening Scales, PTSD Checklist for *Diagnostic and Statistical Manual of Mental Disorders, Fifth Edition*, and Columbia-Suicide Severity Rating Scale. Logistic regression was used to estimate associations of bullying or hazing exposure with the outcomes.

**Results:**

The 1463 participants were predominantly male (weighted percentage [SE], 90.4% [0.9%]) and had a mean (SE) age of 21.1 (0.1) years at baseline. At STARRS-LS1, 188 respondents (weighted percentage [SE], 12.2% [1.1%]) reported bullying or hazing during deployment. Weighted outcome prevalences were 18.7% (1.3%) for MDD, 5.2% (0.9%) for intermittent explosive disorder, 21.8% (1.5%) for PTSD, 14.2% (1.2%) for suicidal ideation, and 8.7% (1.0%) for SUD. In models that adjusted for baseline sociodemographic and clinical characteristics and other potential traumas, exposure to bullying or hazing was significantly associated with MDD (adjusted odds ratio [aOR], 2.92; 95% CI, 1.74-4.88), intermittent explosive disorder (aOR, 2.59; 95% CI, 1.20-5.59), PTSD (aOR, 1.86; 95% CI, 1.23-2.83), suicidal ideation (aOR, 1.91; 95% CI, 1.17-3.13), and SUD (aOR, 2.06; 95% CI, 1.15-3.70).

**Conclusions and Relevance:**

In this cohort study of combat-deployed soldiers, reports of being bullied or hazed during deployment were associated with mental disorders and suicidal thoughts. Recognition of these associations may inform efforts to prevent and address mental health problems among service members.

## Key Points

**Question:**

Is being bullied or hazed by fellow unit members during a combat deployment associated with poorer mental health outcomes among US Army soldiers?

**Findings:**

This cohort study analyzed data from 1463 combat-deployed soldiers and found that reports of being bullied or hazed during deployment were significantly associated with major depressive disorder, intermittent explosive disorder, posttraumatic stress disorder, suicidal thoughts, and substance use disorder.

**Meaning:**

Recognition of the associations between bullying or hazing and mental health conditions can inform efforts to prevent and address these problems in combat-deployed service members.

## Teaser

This cohort study of US Army soldiers assesses whether bullying or hazing during deployment is associated with mental health outcomes.

## Introduction

Workplace bullying encompasses a variety of threatening, humiliating, and disruptive acts that occur in an occupational setting and are intended to cause physical or psychological harm to the person being bullied.^1^ The related phenomenon of hazing involves similar behaviors but with the purported aim of initiating the person being hazed into a group. Prospective studies^2,3,4,5,6,7^ indicate that workplace bullying is associated with onset of mental health problems in civilians. However, few studies have examined associations between bullying or hazing and mental health outcomes among military personnel.

The available data from military samples suggest links between bullying or hazing exposure and problems such as depression, anger, alcohol misuse, and suicidal thoughts.^8,9,10,11,12^ However, the extent to which the observed associations reflect influences of concomitant risk factors (eg, preexisting mental health problems or other stressors) is poorly understood. Investigation of whether exposure to bullying or hazing explains unique variance in mental health outcomes of service members could inform efforts to reduce the burden of mental disorders and suicidality in this population.

In support of this aim, the current study examines associations of bullying or hazing with mental health outcomes among combat-deployed soldiers. Deployment is a period characterized by increased stress and diminished access to nonmilitary sources of social support that may protect against adverse outcomes of bullying or hazing.^13^ Unit cohesion appears to mitigate the effects of deployment stressors^14^; however, this buffer may be compromised for soldiers who are targets of malicious behavior perpetrated by fellow unit members. Of importance, whereas stressors such as combat are unavoidable for some deployed soldiers, bullying and hazing can be prevented and/or addressed by leaders (ie, they can be intervention targets). These contextual factors provide additional impetus for examining associations between mental health and exposure to bullying or hazing in a deployment setting.

The analyses described in this report specifically evaluate associations between bullying or hazing during deployment and major depressive disorder (MDD), intermittent explosive disorder, posttraumatic stress disorder (PTSD), suicidal ideation, and substance use disorder (SUD) in wave 1 of the Study to Assess Risk and Resilience in Servicemembers (STARRS) Longitudinal Study (STARRS-LS1).^15^ The analyses estimate these associations controlling for sociodemographic and clinical characteristics measured in the baseline New Soldier Study (NSS),^16^ as well as other potential traumas experienced by soldiers during the follow-up interval. Given the distinctive aspects of bullying or hazing vis-à-vis other military stressors, the hypothesis of this study was that bullying or hazing during deployment would be independently associated with the mental health outcomes.

## Methods

### Overview and Participants

STARRS-LS recruited a probability sample of soldiers who had participated in components of Army STARRS^16^ while on active duty. The first wave of follow-up data collection (STARRS-LS1) occurred from September 1, 2016, to April 30, 2018, with surveys administered via web or telephone (weighted response rate, 35.6%). Data were analyzed from October 11, 2021, to October 28, 2022. STARRS-LS1 participants provided written informed consent, and the study was approved by the human subjects committees of the collaborating institutions. Other information regarding STARRS-LS1 and the study measures is provided in the eMethods in Supplement 1 and a prior publication.^15^ This study followed the Strengthening the Reporting of Observational Studies in Epidemiology (STROBE) reporting guideline.

This study focuses on the subset of the STARRS-LS1 cohort whose baseline was the Army STARRS NSS.^17^ The NSS was conducted at 3 US Army installations from April 1, 2011, to November 30, 2012. Consenting soldiers completed a computerized survey before Basic Combat Training that assessed sociodemographic characteristics, lifetime *Diagnostic and Statistical Manual of Mental Disorders, Fourth Edition* mental disorders, and risk/protective factors. The final NSS sample^18^ consisted of 38 507 soldiers, 6216 of whom completed the STARRS-LS1 survey. Of these, 1467 reported ever deploying to a combat theater, making them eligible for this study. Four eligible respondents were excluded due to missing bullying or hazing data, resulting in a sample size of 1463. The weighted mean (SE) interval between NSS and STARRS-LS1 surveys was 5.4 (0.03) years. The eMethods in Supplement 1 contains additional information about the study measures.

### Measures

#### Outcomes

The STARRS-LS1 survey evaluated *Diagnostic and Statistical Manual of Mental Disorders, Fifth Edition* mental disorders with items adapted from the Composite International Diagnostic Interview Screening Scales (CIDI-SC)^19^ and PTSD Checklist for *DSM-5* (PCL-5).^20^ Suicidal ideation was assessed using an expanded self-report version of the Columbia-Suicide Severity Rating Scale (C-SSRS).^21^ The outcomes for this study were MDD, intermittent explosive disorder, PTSD, and suicidal ideation in the 12 months before STARRS-LS1 and SUD in the 30 days before STARRS-LS1 (SUD in the past 12 months was not assessed).

#### Bullying or Hazing

The STARRS-LS1 survey queried events that occurred “during any deployment you had in a combat theatre since your last interview.” For the NSS cohort, this timeframe covered all the soldier’s deployments to that point. The item assessing bullying or hazing asked if the respondent had been bullied or hazed by 1 or more members of their unit. Response options on the web survey were 0, 1, 2 to 4, 5 to 9, and 10 or more times, whereas options on the telephone interview were yes or no. For harmonization purposes, all responses to the bullying or hazing item were coded as 0 (0 times/no) or 1 (≥1 time/yes).

#### Other Potential Traumas

The STARRS-LS1 survey also assessed exposure to combat stressors, sexual assault during deployment, physical assault during deployment, and potential traumas that occurred outside deployments. Six trauma exposure variables were derived for logistic regression analysis: a combat exposure score (range, 0-11, with 0 indicating no reported exposure to the combat experiences assessed in the survey and 11 indicating reported exposure to all combat experiences assessed in the survey) and binary variables capturing physical assault (while deployed or not), sexual assault (while deployed or not), other life-threatening events (outside deployment), witnessing or being repeatedly exposed to details of traumas that happened to other people (outside deployment), and serious injury or death of a loved one (outside deployment). Two additional binary variables were derived for descriptive purposes. High combat exposure identified respondents scoring in the upper quartile of the combat exposure score distribution (combat exposure score >5). Noncombat trauma exposure identified respondents reporting any physical assault, any sexual assault, other life-threatening events (outside deployment), witnessing or being exposed to details of trauma that happened to others (outside deployment), and serious injury or death of a loved one (outside deployment).

#### Baseline Mental Health Status and Soldier Characteristics

The NSS survey evaluated lifetime *DSM-IV* mental disorders using items adapted from the CIDI-SC^19^ and the PTSD Checklist–Civilian Version (PCL-C)^22^ and lifetime suicidal ideation using an expanded self-report version of the C-SSRS.^21^ To account for variance in mental health outcomes related to sociodemographic and service characteristics, all multivariable models included age, sex, race and ethnicity, educational level (coded General Educational Development or equivalent, high school diploma, or college degree), and service component (regular US Army, US Army National Guard, or US Army Reserve). These data were collected via self-report in the NSS survey; for race and ethnicity, respondents chose 1 or more options from predetermined categories. Given low endorsement of certain categories, race and ethnicity responses were recoded as Hispanic, non-Hispanic Black, non-Hispanic White, or other (includes American Indian or Alaska Native, Asian, Native Hawaiian or other Pacific Islander, and any other race).

### Statistical Analysis

All statistical analyses were conducted in R, version 3.6.1 (R Foundation for Statistical Computing).^23^ Weights developed for the NSS and STARRS-LS1 surveys were applied to adjust for nonresponse and for differences between respondents and the population of soldiers they were intended to represent (ie, poststratification weights). Details about the weights are provided elsewhere.^15,24^ Differences between respondents who reported vs denied being bullied or hazed during deployment were evaluated using design-based Wald tests and 2-sided, unpaired *t* tests for categorical and continuous variables, respectively. Weights-adjusted logistic regression was used to estimate the associations of exposure to bullying or hazing during deployment with mental health outcomes at STARRS-LS1. Subsequent logistic regression models estimated these associations adjusting for sociodemographic and service characteristics and lifetime history of the outcome at baseline (note that the model of suicidal ideation also adjusted for lifetime MDD at baseline). Final models added adjustment for other potential traumas during the follow-up interval (n = 1431 for these models because 32 respondents were missing data for 1 or more trauma variables). Two-tailed *P* < .05 was considered statistically significant.

## Results

The sample comprised 1463 soldiers, of whom 1269 were male (weighted percentage [SE], 90.4% [0.9%]) and 194 were female (weighted percentage [SE], 9.6% [0.9%]), with a mean (SE) age of 21.1 (0.1) years. A total of 222 respondents were Hispanic (weighted percentage [SE], 18.3% [1.3%]), 185 were Black (weighted percentage [SE], 16.2% [1.2%]), 945 were White (weighted percentage [SE], 58.1% [1.7%]), and 111 were of other race or ethnicity (weighted percentage [SE], 7.4% [0.9%]). A majority of respondents had enlisted in the regular Army (n = 1104; weighted percentage [SE], 80.4% [1.6%]). Table 1 lists the other characteristics of the sample and of the 188 respondents (weighted percentage [SE], 12.2% [1.1%]) who reported that they had been bullied or hazed during a combat deployment. Respondents who reported bullying or hazing during deployment were younger, disproportionately female, more likely to have reported lifetime PTSD and suicidal ideation at baseline, and more likely to have reported several other deployment and nondeployment stressors (Table 1). The Figure depicts the co-occurrence of bullying or hazing during deployment with other types of trauma exposure during the follow-up interval.

**Table 1.** Characteristics of the Total Sample and the Group Exposed to Bullying or Hazing During Deployment^a^

Characteristic	Total sample (N = 1463)		Bullied or hazed during deployment (n = 188)		Exposed vs nonexposed	
	Unweighted No.	Weighted % (SE)	Unweighted No.	Weighted % (SE)	F	*P* value
Sex						
Male	1269	90.4 (0.9)	151	84.5 (2.8)	6.23	.01
Female	194	9.6 (0.9)	37	15.5 (2.8)		
Age at baseline, weighted mean (SE), y	NA	21.1 (0.1)	NA	20.6 (0.2)	−2.23	.03
Race and ethnicity						
Hispanic	222	18.3 (1.3)	29	16.6 (3.5)	0.15	.93
Non-Hispanic						
Black	185	16.2 (1.2)	21	17.3 (4.5)		
White	945	58.1 (1.7)	122	57.8 (4.5)		
Other race and ethnicity^b^	111	7.4 (0.9)	16	8.4 (2.3)		
Educational level						
High school diploma	1114	80.9 (1.2)	149	84.7 (2.9)	1.29	.28
GED or equivalent	143	9.8 (0.9)	14	6.6 (2.1)		
College degree	206	9.3 (0.8)	25	8.7 (2.3)		
Service component						
Regular US Army	1104	80.4 (1.6)	147	85.1 (2.8)	1.67	.19
US Army National Guard	224	13.8 (1.3)	27	10.9 (2.5)		
US Army Reserve	135	5.8 (0.7)	14	3.9 (1.2)		
Mental health condition						
Lifetime major depressive disorder at baseline	106	5.6 (0.6)	20	8.7 (2.3)	2.02	.16
Lifetime intermittent explosive disorder at baseline^c^	253	13.3 (0.9)	37	15.3 (2.9)	0.44	.51
Lifetime posttraumatic stress disorder at baseline	196	10.4 (1.0)	39	22.2 (4.0)	9.96	.002
Lifetime suicidal ideation at baseline	276	11.3 (0.7)	52	20.0 (3.3)	7.64	.007
Lifetime substance use disorder at baseline	232	11.6 (0.7)	26	11.6 (2.4)	0.00	.99
Exposure						
Combat exposure, weighted mean (SE)^d^	NA	3.4 (0.1)	NA	4.2 (0.2)	3.24	.002
Physical assault since baseline^e^	51	3.4 (0.6)	16	8.0 (2.5)	4.50	.04
Sexual assault since baseline^f^	48	2.3 (0.4)	21	7.9 (2.0)	10.47	.002
Other life-threatening event since baseline^g^	298	20.3 (1.5)	61	30.4 (4.9)	5.17	.03
Exposed to others’ trauma since baseline^g^	403	30.5 (1.7)	72	40.1 (4.6)	5.10	.03
Injury or death of loved one since baseline^g^	263	19.5 (1.4)	48	26.2 (5.0)	2.43	.12

Abbreviations: GED, General Educational Development; NA, not applicable (characteristic is a continuous variable).

^a^ Data are presented as indicated in the column headings except for age at baseline and combat exposure, which are presented as weighted mean (SE) with a *t* value rather than an F value reported.

^b^ Includes Asian, American Indian/Alaska Native, Native Hawaiian/other Pacific Islander, and other. Respondents selecting *other* were given an opportunity to provide their own description of their race and ethnicity.

^c^ Due to differences in the *Diagnostic and Statistical Manual of Mental Disorders, Fourth Edition* (baseline) and *Diagnostic and Statistical Manual of Mental Disorders, Fifth Edition* (follow-up) definitions of intermittent explosive disorder, this estimate is not directly comparable to the prevalence estimate for 12-month intermittent explosive disorder at the Study to Assess Risk and Resilience in Servicemembers Longitudinal Study wave 1 follow-up (reported in Results).

^d^ The range of the combat exposure score was 0 to 11, with 0 indicating no reported exposure to the combat experiences assessed in the survey and 11 indicating reported exposure to all combat experiences assessed in the survey. Twenty-seven respondents were missing responses on 1 or more combat exposure items; thus, the reported estimates are based on sample sizes of 1436 in the total sample and 181 in the bullied or hazed group.

^e^ Three respondents were missing responses to 1 or both physical assault items; thus, the reported estimate is based on sample sizes of 1460 in the total sample and 187 in the bullied or hazed group. Prevalence was higher in the bullied or hazed group vs the nonexposed group for physical assaults that occurred during deployment (3.9% vs 1.1%; F = 3.65, *P* = .06) as well as outside deployment (5.8% vs 1.9%; F = 3.33, *P* = .07).

^f^ Three respondents were missing responses to 1 or both sexual assault items (none of whom reported bullying or hazing exposure); thus, the reported estimate is based on a total sample size of 1460. Prevalence was significantly higher in the bullied or hazed group vs the nonexposed group for sexual assaults that occurred during deployment (5.7% vs 0.7%; F = 9.06, *P* = .003) as well as outside deployment (5.1% vs 1.3%; F = 7.94, *P* = .006).

^g^ Refers to events that occurred outside deployment periods.

**Figure.**
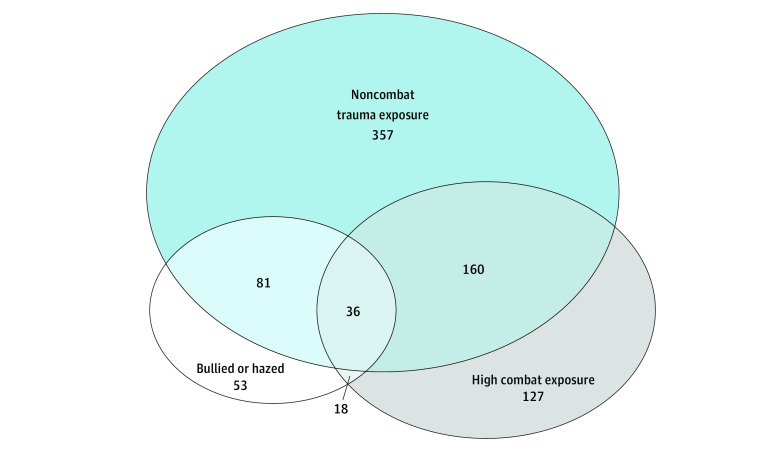
Co-occurrence of Bullying or Hazing Exposure During Deployment and Other Postenlistment Trauma Exposure The sizes of the circles and overlapping areas are proportional to the number of participants reporting the indicated exposure(s).

At the STARRS-LS1 follow-up, weighted percentages (SEs) of 42.1% (1.6%) of respondents were on active duty in the regular US Army, 4.7% (0.7%) were activated guard members or reservists, 21.2% (1.5%) were deactivated guard members or reservists, 27.9% (1.3%) were separated, and 4.1% (0.7%) were retired. Outcome prevalences were 18.7% (1.3%) for MDD, 5.2% (0.9%) for intermittent explosive disorder, 21.8% (1.5%) for PTSD, 14.2% (1.2%) for suicidal ideation, and 8.7% (1.0%) for SUD. Table 2 summarizes the results of logistic regression models that estimated associations between exposure to bullying or hazing during deployment and the outcomes. In unadjusted models, bullying or hazing was associated with significantly increased risk of all the mental health outcomes (OR, 3.60 [95% CI, 2.33-5.57] for MDD in the past 12 months; OR, 3.32 [95% CI, 1.40-7.84] for intermittent explosive disorder in the past 12 months; OR, 2.99 [95% CI, 2.04-4.38] for PTSD in the past 12 months; OR, 2.74 [95% CI, 1.83-4.09] for suicidal ideation in the past 12 months; and OR, 2.52 [95% CI, 1.30-4.88] for SUD in the past 30 days) (Table 2). Some attenuation of these associations occurred when controls were added for baseline sociodemographic and clinical characteristics and for other potential traumas that had occurred during the follow-up interval. However, as indicated in Table 2, reports of bullying or hazing during deployment remained significantly associated with all outcomes, even in the fully adjusted models. Detailed results of the fully adjusted models are provided in eTables 1 to 5 in Supplement 1.

**Table 2.** Associations of Exposure to Bullying or Hazing During Deployment With Mental Health Outcomes at the Study to Assess Risk and Resilience in Servicemembers Longitudinal Study Wave 1 Follow-up

Outcome	Odds ratios for bullying or hazing exposure (95% CI)		
	Unadjusted	Adjusted for baseline factors^a^	Adjusted for baseline factors and other trauma exposures^b^
Major depressive disorder in the past 12 mo	3.60 (2.33-5.57)^c^	3.61 (2.33-5.60)^c^	2.92 (1.74-4.88)^c^
Intermittent explosive disorder in the past 12 mo	3.32 (1.40-7.84)^d^	3.34 (1.44-7.76)^d^	2.59 (1.20-5.59)^e^
Posttraumatic stress disorder in the past 12 mo	2.99 (2.04-4.38)^c^	2.47 (1.66-3.67)^c^	1.86 (1.23-2.83)^d^
Suicidal ideation in the past 12 mo	2.74 (1.83-4.09)^c^	2.55 (1.66-3.92)^c^	1.91 (1.17-3.13)^e^
Substance use disorder in the past 30 d	2.52 (1.30-4.88)^d^	2.62 (1.38-4.98)^d^	2.06 (1.15-3.70)^e^

^a^ Age, sex, race and ethnicity, educational level, service component, and lifetime history of the specified mental health condition as reported in the baseline survey (New Soldier Study). In addition to these characteristics, the model of suicidal ideation adjusted for lifetime major depressive disorder at baseline.

^b^ Combat stressors (scale, 0-11, with 0 indicating no reported exposure to the combat experiences assessed in the survey and 11 indicating reported exposure to all combat experiences assessed in the survey), physical assault, sexual assault, other life-threatening experiences, serious injury or death of a close loved one, and witnessing or being repeatedly exposed to details of trauma that happened to other people. Sample size was 1431 for the models that adjusted for other trauma exposures because 32 respondents were missing data for 1 or more of these variables.

^c^ *P* < .001.

^d^ *P* < .01.

^e^ *P* < .05.

## Discussion

In this cohort study of combat-deployed soldiers, reports of having been bullied or hazed by fellow unit members during deployment were associated with mental disorders and suicidal ideation at the STARRS-LS1 follow-up. These associations partly reflected that bullying or hazing and other potential traumas (eg, combat exposure and sexual assault) were highly intercorrelated and explained common variance in mental health outcomes. Nevertheless, bullying or hazing during deployment displayed independent associations with all study outcomes, including MDD, intermittent explosive disorder, PTSD, suicidal ideation, and SUD. Although causality cannot be assumed, these results raise the possibility that US Army policies and programs (eg, leader trainings) that aim to eradicate bullying and hazing may help reduce mental disorders and suicidality among soldiers.

Bullying or hazing displayed a particularly strong association with MDD in the past 12 months at the STARRS-LS1 follow-up. Interpersonal problems are a common antecedent of MDD,^25^ and aspects of bullying or hazing (eg, social rejection, humiliation) might trigger or exacerbate depressed mood in vulnerable individuals.^26,27^ Other data suggest that depressed mood may at times elicit social rejection or relationship stress,^28^ potentially fostering a vicious cycle in which interpersonal problems and depression worsen over time. Although our models controlled for lifetime MDD at baseline, depression may have predated the bullying or hazing in some cases (eg, subthreshold MDD may have been present at baseline; onset of a depressive disorder could have occurred during the interval between baseline and exposure to bullying or hazing during deployment). Thus, the association between bullying or hazing during deployment and MDD is likely attributable to diverse and potentially bidirectional mechanisms.

Bullying or hazing during deployment also exhibited a strong association with intermittent explosive disorder in the past 12 months, a condition characterized by aggressive outbursts. This finding is broadly consistent with cross-national epidemiologic data showing elevated rates of intermittent explosive disorder among individuals exposed to interpersonal violence.^29^ As with MDD, various mechanisms could underlie the association between bullying or hazing and intermittent explosive disorder.^8,9^ Perceived injustice is a key anger trigger; as such, it is plausible that the experience or subsequent recollection of bullying or hazing could lead to increased anger or lower the threshold for aggressive outbursts. Conversely, preexisting anger difficulties (which may not have been fully captured in our models) could contribute to aggressive exchanges with unit members, which in turn could affect the likelihood of being a target of bullying or hazing.

Exposure to bullying or hazing also explained unique variance in PTSD, suicidal ideation, and SUD. Of note, the survey did not ascertain whether PTSD symptoms were triggered by bullying or hazing. Indeed, many forms of bullying or hazing do not involve actual or threatened physical injury or sexual violence (ie, they would not meet criterion A for *DSM-5* diagnosis of PTSD). Nevertheless, contextual factors may exacerbate or mitigate impacts of traumatic events. Perceiving an atmosphere of threat within one’s unit could increase a soldier’s vulnerability to PTSD following other traumas, much like perceiving support from fellow unit members appears to protect against PTSD in combat-exposed soldiers.^14^ The association of bullying or hazing with suicidal ideation likely relates to the elevated rate of MDD in exposed soldiers. Some evidence also suggests that perceptions of being a burden to other people may contribute to associations between bullying exposure and suicidality.^12^ The observation of elevated risk of SUD among combat-deployed soldiers exposed to bullying or hazing converges with results showing greater alcohol misuse among South Korean military personnel exposed to hazing^9^ and is consistent with evidence that substance use may be used as a way of coping with negative emotions that can result from workplace exposure to bullying or hazing.^30^

Approximately 1 in 8 soldiers (12.2%) reported that they had been bullied or hazed while deployed, suggesting the eradication of these behaviors could impact large numbers of service members during a critical time. Although investigations of unit-level influences on bullying or hazing are scarce, a study^31^ of Norwegian naval personnel found that departments receiving higher scores on a measure of fair leadership had lower proportions of personnel reporting that they had experienced bullying or witnessed a fellow unit member being bullied. Fair leadership included behaviors such as superiors treating members fairly and equally or distributing duties in a fair and equitable manner. It may be worthwhile to evaluate whether training focused on cultivating fair leadership practices affect incidence of bullying or hazing within military units.

Several sociodemographic and clinical correlates of bullying or hazing observed in this combat-deployed population have been reported in prior studies.^31,32,33,34,35^ For example, civilian studies have found workplace bullying to be more commonly reported by women^32,33^ and those with preexisting PTSD,^34^ implying that these characteristics are associated with reports of being bullied across different occupational settings, not just in the context of military deployment. Another study^31^ found that younger military personnel were more likely to have experienced or witnessed bullying in their unit, converging with our results indicating a higher rate of being bullied among younger soldiers. Finally, in this study, bullying or hazing was associated with more combat exposure, which may indicate that these behaviors are more common in units with more direct combat roles. A study^35^ of female UK military veterans similarly found that military adversity, which included emotional bullying, was more frequently reported by women who had served in combat or combat support roles. To help target prevention efforts, future studies should evaluate whether bullying and hazing tend to cluster within military units and whether certain unit characteristics (eg, combat role, cohesion, and leadership) are associated with incidence of bullying or hazing.

Finally, soldiers who reported being bullied or hazed were also more likely to report physical and sexual assault (including during deployment). The survey did not clarify whether those traumas occurred separately or as part of the bullying or hazing.^36^ The survey also did not assess other forms of military sexual trauma (eg, sexual harassment) that may co-occur or have unclear boundaries with bullying or hazing. Future studies should gather more detailed information to elucidate the unique vulnerabilities of individuals who experience distinct types of interpersonal trauma (eg, physical aggression or verbal abuse) or bullying or hazing that involves sexually assaultive or harassing behavior. The co-occurrence of bullying or hazing with combat exposure and noncombat trauma is consistent with evidence that stressors may cluster together and compound risk for some individuals.^37,38^

### Limitations

This study has some limitations. First, causal inferences should not be made, because mental health conditions observed at follow-up could have predated the bullying or hazing exposure and unmeasured variables might have contributed to the observed associations between bullying or hazing exposure and the outcomes. We mitigated these issues by controlling for baseline characteristics (including lifetime history of mental health conditions) and other trauma exposure; however, these issues remain limitations of the study. Second, retrospective reports of life events and mental health symptoms may be affected by inaccurate recall, hesitancy to report stigmatized experiences, and reporting biases. Possible effects include underestimation of the prevalence of bullying or hazing or other abuse due to poor recall or underreporting of stigmatized experiences and potential inflation of associations between bullying or hazing and mental health problems due to mood-congruent reporting biases (eg, negative emotions could facilitate recall of adverse events, whereas positive emotions could foster minimization of bullying or hazing experiences). Third, the survey included only 1 item that assessed bullying or hazing exposure. Definitions of these exposures were not provided, and participants’ self-reports were not validated through other modalities. Respondents may have reported events that would not meet accepted definitions of bullying or hazing or failed to report bullying or hazing because they were unsure if their experiences qualified as such. Fourth, the nature of bullying or hazing was not assessed (including whether it involved sexual assault or harassment), and its frequency was not considered because telephone participants were only administered a yes or no item about bullying or hazing. Therefore, we were unable to evaluate how the nature or frequency of the bullying or hazing affected risk of mental health problems. Moreover, although bullying and hazing are similar phenomena, there may be differences in their psychological impact or in risk factors for being a target of bullying vs hazing. Then again, discriminating between bullying and hazing is often not possible because the intent of the malicious behavior and how the target interprets it may diverge (or be ambiguous).^11^ Finally, some individuals may have experienced mental health problems related to bullying or hazing that were not captured in the STARRS-LS1 survey (eg, if the problems occurred during or shortly after deployment and resolved >1 year before the follow-up survey).

## Conclusions

In this cohort study of combat-deployed US Army soldiers, reports of being bullied or hazed during deployment were associated with mental disorders and suicidal ideation at follow-up. These associations remained significant after adjusting for baseline characteristics and other potential traumas during the follow-up period. Unlike combat exposure, bullying or hazing is an avoidable event that appears to affect a substantial proportion of deployed soldiers (approximately 1 in 8 in this sample). Continued vigilance and implementation of prevention strategies^39^ is warranted and may help reduce incidence of mental health problems among soldiers. Furthermore, fostering awareness and effective responses among unit leaders is important when bullying or hazing occurs, given evidence that support from leadership may buffer some effects of peer abuse.^40^ Finally, more research is needed to replicate these findings and clarify how the nature, frequency, and timing of bullying or hazing relate to mental health risk.

## Notes

Supplement 1. **eMethods.** Supplemental Methods **eTable 1.** Results of the Fully Adjusted Model of Past-12-Month Major Depressive Disorder **eTable 2.** Results of the Fully Adjusted Model of Past-12-Month Intermittent Explosive Disorder **eTable 3.** Results of the Fully Adjusted Model of Past-12-Month Posttraumatic Stress Disorder **eTable 4.** Results of the Fully Adjusted Model of Past-12-Month Suicidal Ideation **eTable 5.** Results of the Fully Adjusted Model of Past-30-Day Substance Use Disorder
eReferences

Supplement 2.
Data Sharing Statement

## References

[1] Keller KM, Matthews M, Hall KC, Marcellino W, Mauro JA, Lim N. Hazing in the U.S. Armed Forces: Recommendations for Hazing Prevention Policy and Practice. RAND Corporation; 2015. Accessed August 16, 2022. https://www.rand.org/pubs/research_reports/RR941.html

[2] Einarsen S, Nielsen MB. Workplace bullying as an antecedent of mental health problems: a five-year prospective and representative study. Int Arch Occup Environ Health. 2015;88(2):131-142. doi:10.1007/s00420-014-0944-7 24840725

[3] Nielsen MB, Nielsen GH, Notelaers G, Einarsen S. Workplace bullying and suicidal ideation: a 3-wave longitudinal Norwegian study. Am J Public Health. 2015;105(11):e23-e28. doi:10.2105/AJPH.2015.302855 26378852 PMC4605166

[4] Gullander M, Hogh A, Hansen AM, . Exposure to workplace bullying and risk of depression. J Occup Environ Med. 2014;56(12):1258-1265. doi:10.1097/JOM.0000000000000339 25479295

[5] Lahelma E, Lallukka T, Laaksonen M, Saastamoinen P, Rahkonen O. Workplace bullying and common mental disorders: a follow-up study. J Epidemiol Community Health. 2012;66(6):e3. doi:10.1136/jech.2010.115212 21252256

[6] Lange S, Burr H, Rose U, Conway PM. Workplace bullying and depressive symptoms among employees in Germany: prospective associations regarding severity and the role of the perpetrator. Int Arch Occup Environ Health. 2020;93(4):433-443. doi:10.1007/s00420-019-01492-7 31781901 PMC7118039

[7] Rodríguez-Muñoz A, Moreno-Jiménez B, Sanz-Vergel AI. Reciprocal relations between workplace bullying, anxiety, and vigor: a two-wave longitudinal study. Anxiety Stress Coping. 2015;28(5):514-530. doi:10.1080/10615806.2015.1016003 25665500

[8] Kim J, Kim J, Park S. Military hazing and suicidal ideation among active duty military personnel: serial mediation effects of anger and depressive symptoms. J Affect Disord. 2019;256:79-85. doi:10.1016/j.jad.2019.05.060 31158719

[9] Kim JY, Kim J, Park S, Fear N. Workplace victimization and alcohol misuse among junior military personnel: mediating the role of anger. J Affect Disord. 2021;294:638-644. doi:10.1016/j.jad.2021.07.010 34332364

[10] Hourani LL, Williams J, Lattimore PK, . Workplace victimization risk and protective factors for suicidal behavior among active duty military personnel. J Affect Disord. 2018;236:45-51. doi:10.1016/j.jad.2018.04.095 29715608

[11] Steele NM, Rodgers B, Fogarty GJ. The relationships of experiencing workplace bullying with mental health, affective commitment, and job satisfaction: application of the job demands control model. Int J Environ Res Public Health. 2020;17(6):2151. doi:10.3390/ijerph17062151 32213864 PMC7143050

[12] Crowell-Williamson GA, Fruhbauerova M, DeCou CR, Comtois KA. Perceived burdensomeness, bullying, and suicidal ideation in suicidal military personnel. J Clin Psychol. 2019;75(12):2147-2159. doi:10.1002/jclp.22836 31332803 PMC11000627

[13] Nielsen MB, Christensen JO, Finne LB, Knardahl S. Workplace bullying, mental distress, and sickness absence: the protective role of social support. Int Arch Occup Environ Health. 2020;93(1):43-53. doi:10.1007/s00420-019-01463-y 31342156

[14] Campbell-Sills L, Flynn PJ, Choi KW, . Unit cohesion during deployment and post-deployment mental health: is cohesion an individual- or unit-level buffer for combat-exposed soldiers? Psychol Med. 2022;52(1):121-131. doi:10.1017/S0033291720001786 32517825 PMC9341401

[15] Stanley IH, Chu C, Gildea SM, . Predicting suicide attempts among U.S. Army soldiers after leaving active duty using information available before leaving active duty: results from the Study to Assess Risk and Resilience in Servicemembers-Longitudinal Study (STARRS-LS). Mol Psychiatry. 2022;27(3):1631-1639. doi:10.1038/s41380-021-01423-4 35058567 PMC9106812

[16] Ursano RJ, Colpe LJ, Heeringa SG, Kessler RC, Schoenbaum M, Stein MB; Army STARRS collaborators. The Army Study to Assess Risk and Resilience in Servicemembers (Army STARRS). Psychiatry. 2014;77(2):107-119. doi:10.1521/psyc.2014.77.2.107 24865195 PMC4075436

[17] Kessler RC, Colpe LJ, Fullerton CS, . Design of the Army Study to Assess Risk and Resilience in Servicemembers (Army STARRS). Int J Methods Psychiatr Res. 2013;22(4):267-275. doi:10.1002/mpr.1401 24318217 PMC3992857

[18] Rosellini AJ, Heeringa SG, Stein MB, . Lifetime prevalence of *DSM-IV* mental disorders among new soldiers in the U.S. Army: results from the Army Study to Assess Risk and Resilience in Servicemembers (Army STARRS). Depress Anxiety. 2015;32(1):13-24. doi:10.1002/da.22316 25338841 PMC5111824

[19] Kessler RC, Ustün TB. The World Mental Health (WMH) Survey Initiative version of the World Health Organization (WHO) Composite International Diagnostic Interview (CIDI). Int J Methods Psychiatr Res. 2004;13(2):93-121. doi:10.1002/mpr.168 15297906 PMC6878592

[20] Blevins CA, Weathers FW, Davis MT, Witte TK, Domino JL. The Posttraumatic Stress Disorder Checklist for *DSM-5* (PCL-5): development and initial psychometric evaluation. J Trauma Stress. 2015;28(6):489-498. doi:10.1002/jts.22059 26606250

[21] Posner K, Brown GK, Stanley B, . The Columbia-Suicide Severity Rating Scale: initial validity and internal consistency findings from three multisite studies with adolescents and adults. Am J Psychiatry. 2011;168(12):1266-1277. doi:10.1176/appi.ajp.2011.10111704 22193671 PMC3893686

[22] Weathers F, Litz B, Herman D, Huska J, Keane T. The PTSD Checklist (PCL): reliability, validity, and diagnostic utility. Poster presented at: International Society for Traumatic Stress Studies; January 1, 1993; San Antonio, Texas.

[23] *R: A Language and Environment for Statistical Computing*. R Foundation for Statistical Computing; 2022. Accessed January 4, 2023. http://www.R-project.org/

[24] Kessler RC, Heeringa SG, Colpe LJ, . Response bias, weighting adjustments, and design effects in the Army Study to Assess Risk and Resilience in Servicemembers (Army STARRS). Int J Methods Psychiatr Res. 2013;22(4):288-302. doi:10.1002/mpr.1399 24318218 PMC3992816

[25] Hammen C. Stress and depression. Annu Rev Clin Psychol. 2005;1:293-319. doi:10.1146/annurev.clinpsy.1.102803.143938 17716090

[26] Kendler KS, Hettema JM, Butera F, Gardner CO, Prescott CA. Life event dimensions of loss, humiliation, entrapment, and danger in the prediction of onsets of major depression and generalized anxiety. Arch Gen Psychiatry. 2003;60(8):789-796. doi:10.1001/archpsyc.60.8.789 12912762

[27] Slavich GM, Irwin MR. From stress to inflammation and major depressive disorder: a social signal transduction theory of depression. Psychol Bull. 2014;140(3):774-815. doi:10.1037/a0035302 24417575 PMC4006295

[28] Liu RT, Alloy LB. Stress generation in depression: a systematic review of the empirical literature and recommendations for future study. Clin Psychol Rev. 2010;30(5):582-593. doi:10.1016/j.cpr.2010.04.010 20478648 PMC3049314

[29] Scott KM, Lim CC, Hwang I, . The cross-national epidemiology of *DSM-IV* intermittent explosive disorder. Psychol Med. 2016;46(15):3161-3172. doi:10.1017/S0033291716001859 27572872 PMC5206971

[30] Aquino K, Thau S. Workplace victimization: aggression from the target’s perspective. Annu Rev Psychol. 2009;60:717-741. doi:10.1146/annurev.psych.60.110707.163703 19035831

[31] Magerøy N, Lau B, Riise T, Moen BE. Association of psychosocial factors and bullying at individual and department levels among naval military personnel. J Psychosom Res. 2009;66(4):343-351. doi:10.1016/j.jpsychores.2008.10.009 19302893

[32] Chan CMH, Wong JE, Yeap LLL, Wee LH, Jamil NA, Swarna Nantha Y. Workplace bullying and psychological distress of employees across socioeconomic strata: a cross-sectional study. BMC Public Health. 2019;19(suppl 4):608. doi:10.1186/s12889-019-6859-1 31196025 PMC6565541

[33] Feijó FR, Gräf DD, Pearce N, Fassa AG. Risk factors for workplace bullying: a systematic review. Int J Environ Res Public Health. 2019;16(11):1945. doi:10.3390/ijerph16111945 31159344 PMC6603960

[34] Nielsen MB, Birkeland MS, Hansen MB, Knardahl S, Heir T. Victimization from workplace bullying after a traumatic event: time-lagged relationships with symptoms of posttraumatic stress. Int Arch Occup Environ Health. 2017;90(5):411-421. doi:10.1007/s00420-017-1204-4 28161885

[35] Hendrikx LJ, Williamson V, Murphy D. Adversity during military service: the impact of military sexual trauma, emotional bullying and physical assault on the mental health and well-being of women veterans. BMJ Mil Health. 2021;bmjmilitary-2021-001948. doi:10.1136/bmjmilitary-2021-001948 34697241

[36] Galovski TE, Street AE, Creech S, Lehavot K, Kelly UA, Yano EM. State of the knowledge of VA military sexual trauma research. J Gen Intern Med. 2022;37(suppl 3):825-832. doi:10.1007/s11606-022-07580-8 36042078 PMC9481813

[37] Turner HA, Finkelhor D, Ormrod R. Poly-victimization in a national sample of children and youth. Am J Prev Med. 2010;38(3):323-330. doi:10.1016/j.amepre.2009.11.012 20171535

[38] Ford JD, Elhai JD, Connor DF, Frueh BC. Poly-victimization and risk of posttraumatic, depressive, and substance use disorders and involvement in delinquency in a national sample of adolescents. J Adolesc Health. 2010;46(6):545-552. doi:10.1016/j.jadohealth.2009.11.212 20472211

[39] Gillen PA, Sinclair M, Kernohan WG, Begley CM, Luyben AG. Interventions for prevention of bullying in the workplace. Cochrane Database Syst Rev. 2017;1(1):CD009778. doi:10.1002/14651858.CD009778.pub2 28134445 PMC6464940

[40] Clausen T, Conway PM, Burr H, . Does leadership support buffer the effect of workplace bullying on the risk of disability pensioning? an analysis of register-based outcomes using pooled survey data from 24,538 employees. Int Arch Occup Environ Health. 2019;92(7):941-948. doi:10.1007/s00420-019-01428-1 30982156

