# Supplementary material for: Exposure to Bullying or Hazing During Deployment and Mental Health Outcomes Among US Army Soldiers
Source: JAMA Netw Open. 2023 Jan 24;6(1):e2252109. doi: 10.1001/jamanetworkopen.2022.52109 (PMC10408263; doi:10.1001/jamanetworkopen.2022.52109)
Supplement: Supplement 1. — eMethods. Supplemental Methods eTable 1. Results of the Fully Adjusted Model of Past-12-Month Major Depressive Disorder eTable 2. Results of the Fully Adjusted Model of Past-12-Month Intermittent Explosive Disorder eTable 3. Results of the Fully Adjusted Model of Past-12-Month Posttraumatic Stress Disorder eTable 4. Results of the Fully Adjusted Model of Past-12-Month Suicidal Ideation eTable 5. Results of the Fully Adjusted Model of Past-30-Day Substance Use Disorder eReferences [file jamanetwopen-e2252109-s001.pdf]

Supplementary Online Content

Campbell-Sills L, Sun X, Kessler RC, Ursano RJ, Jain S, Stein MB. Exposure to bullying or hazing during deployment and mental health outcomes among US Army soldiers. *JAMA Netw Open*. 2023;6(1):e2252109. doi:10.1001/jamanetworkopen.2022.52109

eMethods. Supplemental Methods

eTable 1. Results of the Fully Adjusted Model of Past-12-Month Major Depressive Disorder

eTable 2. Results of the Fully Adjusted Model of Past-12-Month Intermittent Explosive Disorder

eTable 3. Results of the Fully Adjusted Model of Past-12-Month Posttraumatic Stress Disorder

eTable 4. Results of the Fully Adjusted Model of Past-12-Month Suicidal Ideation

eTable 5. Results of the Fully Adjusted Model of Past-30-Day Substance Use Disorder

eReferences

This supplementary material has been provided by the authors to give readers additional information about their work.

© 2023 Campbell-Sills L et al. *JAMA Network Open.*

eMethods. Supplemental methods

Expanded overview of the STARRS-Longitudinal Study (STARRS-LS)

Soldiers who participated in Army STARRS baseline surveys [New Soldier Study (NSS), All Army Study, or Pre/Post Deployment Study][1] and consented to linkage of their survey data and their Army/ Department of Defense administrative records comprised the eligible sample for STARRS-LS. Given the limited resources for data collection, eligible Army STARRS participants were stratified for differential sampling. Stratum 1 (n=22,176) included those who reported lifetime mental disorders or suicidality in their baseline survey, or who were ever in special operation forces. Stratum 2 (n=26,833) included those who were female or Guard/Reserve members. Stratum 3 (n=23,378) included all remaining baseline survey respondents. The STARRS-LS wave 1 survey (STARRS-LS1) attempted to recruit 100% of Stratum 1 and probability samples of 67% of Stratum 2 and 50% of Stratum 3 for a follow-up. This strategy aligned with the aims of STARRS-LS, which involved a focus on special operation forces, Guard/Reserve, and female soldiers. The STARRS-LS1 survey was conducted from September 2016 to April 2018; eligible participants were given the option of completing either a web-based survey or a telephone interview. A total of 14,508 individuals participated (weighted response rate=35.6%). The weights developed for the survey data incorporate nonresponse and post-stratification adjustments from the baseline Army STARRS surveys and the STARRS-LS1 survey. Details regarding the weights are available elsewhere.[2]

Additional information about the study measures

The Army STARRS and STARRS-LS survey instruments are available at www.starrs-ls.org. The surveys evaluated mental disorders using items adapted from the Composite International Diagnostic Interview Screening Scales,[3] Posttraumatic Stress Disorder (PTSD) Checklist-Civilian version,[4] and PTSD Checklist for DSM-5.[5] Survey-based diagnoses were validated against structured clinical interviews in the Army STARRS Clinical Reappraisal Study.[6] The assessments of mental disorders in the NSS survey (based on DSM-IV criteria) and the STARRS-LS1 survey (based on DSM-5 criteria) were similar except in the case of intermittent explosive disorder. A major revision of the intermittent explosive disorder category for DSM-5 included, among other changes, the addition of a requirement that aggressive outbursts cause clinically significant impairment or distress. To better approximate the DSM-5 definition, we included adjustment for “impairing” DSM-IV intermittent explosive disorder at baseline in the model of DSM-5 intermittent explosive disorder at STARRS-LS1 (see eTable 2 note for details). Suicidal ideation was assessed using an expanded self-report version of the Columbia-Suicide Severity Rating Scale.[7] Suicidal ideation was coded as present if respondents reported having thoughts of killing themselves, or wishing they were dead or would go to sleep and never wake up.

The STARRS-LS1 survey evaluated potential traumas that occurred since the respondent’s last Army STARRS survey. Events that occurred during and outside of deployment periods were queried separately. On the web-based survey, respondents indicated the frequency of exposure to deployment stressors (0, 1, 2-4, 5-9, or 10 or more times). Phone respondents answered a series of yes/no questions about exposure to the deployment stressors. For harmonization purposes, responses to the deployment stress items were coded as 0 (0 times/“no”) or 1 ($\geq$1 time/“yes”). With respect to specific deployment events, the survey assessed combat experiences, physical assault (e.g., mugging), sexual assault, and bullying/hazing victimization. Combat experiences included going on combat patrols; firing rounds or taking enemy fire; getting wounded; having a close call (e.g., equipment shot off body); being responsible for deaths of enemy combatants, non-combatants, or allies; saving lives of service members or civilians; and seeing destroyed homes/villages, severely wounded or dying/dead people, and violence among or maltreatment of non-combatants. Exploratory factor analysis suggested a dominant first factor with item-factor loadings ranging from .68 to .80. Based on this, we combined the items into a summary score. This combat exposure score reflected the number of experiences from the above list that the respondent reported having experienced 1 or more times (theoretical range=0-11).

The STARRS-LS1 survey also asked, “Not counting times during deployment, did you have any of the following stressful experiences since your last survey… serious physical assault (e.g., mugging), sexual assault or rape, life-threatening illness, life-threatening injury, life-threatening accident where you escaped injury (a near miss), natural disaster, any other experience that put you at risk of death or serious injury, serious injury or unexpected death of a close loved one, witnessed someone being seriously injured or killed, discovered or handled a dead body, or exposed to details about highly stressful events as part of your job (e.g., first responders collecting human remains; human services professionals repeatedly being exposed to details about child abuse; medical personnel repeatedly being exposed to death and dying).” Web-based survey respondents checked a box next to each event they had experienced, and phone respondents indicated “yes” or “no” when prompted with each event.

Responses to the above items were used to derive additional trauma exposure variables used in the analysis. *Life-threatening events* included any report of illness, injury, accident without injury (near miss), natural disaster, or another experience that involved a risk of death or serious injury. *Witnessing or being exposed to trauma that happened to other people* included any report of witnessing someone being seriously injured/killed, discovering or handling a dead body, or exposure to trauma details in an occupational setting. *Serious injury or death of a close loved one* was based on a single item that queried this specific experience. Exposure to each of the above trauma categories was coded as present or absent based on whether the respondent had experienced any event in the category at least once.

As previously noted, the STARRS-LS1 survey also assessed physical assault and sexual assault that had occurred since the respondent's last survey (the NSS survey in this case), both during any deployment and outside of deployment periods. Due to the low number of reported events in these categories, we created summary variables capturing any *sexual assault (during or outside of deployments)* and any *physical assault (during or outside of deployments)*, rather than modeling effects of assaults that had happened during versus outside of deployment periods separately.

© 2023 Campbell-Sills L et al. *JAMA Network Open.*

eTable 1
Fully adjusted model of past 12-month major depressive disorder (n=1431)

	Adjusted odds ratio (95% CI)	*p*
Bullied/hazed during deployment	2.92 (1.74-4.88)	<.001
Age in years	1.03 (0.99-1.08)	.19
Female (reference: male)	1.70 (0.96-3.01)	.07
Hispanic (reference: non-Hispanic White)	1.55 (0.88-2.73)	.14
Non-Hispanic Black (reference: non-Hispanic White)	1.14 (0.67-1.93)	.64
Non-Hispanic Other (reference: non-Hispanic White)	1.46 (0.69-3.06)	.32
GED (reference: High School diploma)	1.36 (0.71-2.60)	.36
College degree (reference: High School diploma)	0.42 (0.20-0.89)	.03
National Guard (reference: Regular Army)	0.53 (0.29-0.97)	.04
Reserve (reference: Regular Army)	0.24 (0.13-0.44)	<.001
Lifetime major depressive disorder at baseline	11.54 (6.75-19.75)	<.001
Combat exposure (0-11)	1.10 (1.01-1.20)	.04
Physical assault since baseline survey	3.48 (1.47-8.26)	.006
Sexual assault since baseline survey	1.25 (0.45-3.48)	.67
Other life-threatening event since baseline survey	0.94 (0.54-1.64)	.84
Exposed to traumas of other people since baseline survey	1.51 (0.88-2.58)	.14
Serious injury or death of loved one since baseline survey	2.17 (1.29-3.67)	.005

© 2023 Campbell-Sills L et al. *JAMA Network Open.*

eTable 2
Fully adjusted model of past 12-month intermittent explosive disorder (n=1431)

	Adjusted odds ratio (95% CI)	*p*
Bullied/hazed during deployment	2.59 (1.20-5.59)	.02
Age in years	0.96 (0.85-1.08)	.47
Female (reference: male)	0.42 (0.10-1.80)	.24
Hispanic (reference: non-Hispanic White)	2.61 (1.06-6.43)	.04
Non-Hispanic Black (reference: non-Hispanic White)	3.52 (1.58-7.85)	.003
Non-Hispanic Other (reference: non-Hispanic White)	1.08 (0.30-3.83)	.91
GED (reference: High School diploma)	0.82 (0.18-3.69)	.80
College degree (reference: High School diploma)	0.92 (0.27-3.15)	.90
National Guard (reference: Regular Army)	0.95 (0.38-2.41)	.92
Reserve (reference: Regular Army)	0.06 (0.01-0.43)	.007
Non-impairing lifetime DSM-IV intermittent explosive disorder at baseline (reference: no intermittent explosive disorder)	0.87 (0.29-2.61)	.80
Impairing lifetime DSM-IV intermittent explosive disorder at baseline (reference: no intermittent explosive disorder)	1.79 (0.70-4.58)	.23
Combat exposure (0-11)	1.09 (0.93-1.29)	.30
Physical assault since baseline survey	3.58 (1.12-11.51)	.04
Sexual assault since baseline survey	0.44 (0.07-3.00)	.41
Other life-threatening event since baseline survey	1.79 (0.72-4.43)	.21
Exposed to traumas of other people since baseline survey	1.35 (0.50-3.71)	.56
Serious injury or death of loved one since baseline survey	2.38 (1.04-5.43)	.04

Note. We adjusted for a 3-level lifetime DSM-IV intermittent explosive disorder variable in this model (No intermittent explosive disorder, non-impairing intermittent explosive disorder, or impairing intermittent explosive disorder at baseline). The distinction between non-impairing and impairing DSM-IV intermittent explosive disorder was based on responses to a New Soldier Study survey item that queried how often anger attacks interfered with the respondent’s life. A response of at least “some of the time” was considered indicative of significant impairment. Addition of an impairment criterion aligns the impairing DSM-IV intermittent explosive disorder variable more closely with the DSM-5 definition of intermittent explosive disorder, which was used for outcome assessment in wave 1 of the STARRS Longitudinal Study (STARRS-LS1).

© 2023 Campbell-Sills L et al. *JAMA Network Open.*

eTable 3
Fully adjusted model of past 12-month posttraumatic stress disorder (n=1431)

	Adjusted odds ratio (95% CI)	*p*
Bullied/hazed during deployment	1.86 (1.23-2.83)	.005
Age in years	1.04 (0.99-1.09)	.14
Female (reference: male)	2.09 (1.19-3.65)	.01
Hispanic (reference: non-Hispanic White)	1.77 (1.04-3.01)	.04
Non-Hispanic Black (reference: non-Hispanic White)	0.88 (0.53-1.47)	.63
Non-Hispanic Other (reference: non-Hispanic White)	1.04 (0.55-1.97)	.90
GED (reference: High School diploma)	0.77 (0.35-1.67)	.50
College degree (reference: High School diploma)	0.45 (0.23-0.88)	.02
National Guard (reference: Regular Army)	0.93 (0.52-1.68)	.81
Reserve (reference: Regular Army)	0.57 (0.28-1.15)	.12
Lifetime posttraumatic stress disorder at baseline	7.73 (5.25-11.39)	<.001
Combat exposure (0-11)	1.27 (1.19-1.36)	<.001
Physical assault since baseline survey	1.55 (0.58-4.12)	.39
Sexual assault since baseline survey	1.36 (0.53-3.46)	.52
Other life-threatening event since baseline survey	1.42 (0.96-2.11)	.09
Exposed to traumas of other people since baseline survey	1.43 (0.98-2.08)	.06
Serious injury or death of loved one since baseline survey	2.04 (1.38-3.00)	<.001

© 2023 Campbell-Sills L et al. *JAMA Network Open.*

eTable 4
Fully adjusted model of past 12-month suicidal ideation (n=1431)

	Adjusted odds ratio (95% CI)	*p*
Bullied/hazed during deployment	1.91 (1.17-3.13)	.01
Age in years	1.05 (1.01-1.10)	.03
Female (reference: male)	0.74 (0.38-1.45)	.38
Hispanic (reference: non-Hispanic White)	1.40 (0.88-2.24)	.16
Non-Hispanic Black (reference: non-Hispanic White)	0.93 (0.43-2.04)	.87
Non-Hispanic Other (reference: non-Hispanic White)	0.67 (0.30-1.50)	.34
GED (reference: High School diploma)	0.50 (0.24-1.00)	.05
College degree (reference: High School diploma)	0.53 (0.27-1.03)	.07
National Guard (reference: Regular Army)	1.10 (0.70-1.73)	.67
Reserve (reference: Regular Army)	0.47 (0.24-0.94)	.04
Lifetime suicidal ideation at baseline	2.44 (1.60-3.72)	<.001
Lifetime major depressive disorder at baseline	1.78 (1.03-3.08)	.04
Combat exposure (0-11)	1.11 (1.02-1.21)	.02
Physical assault since baseline survey	1.34 (0.54-3.37)	.53
Sexual assault since baseline survey	3.48 (1.26-9.66)	.02
Other life-threatening event since baseline survey	0.94 (0.54-1.66)	.84
Exposed to traumas of other people since baseline survey	0.95 (0.65-1.39)	.81
Serious injury or death of loved one since baseline survey	1.39 (0.87-2.21)	.17

© 2023 Campbell-Sills L et al. *JAMA Network Open.*

eTable 5
Fully adjusted model of past 30-day substance use disorder (n=1431)

	Adjusted odds ratio (95% CI)	*p*
Bullied/hazed during deployment	2.06 (1.15-3.70)	.02
Age in years	0.91 (0.83-1.00)	.05
Female (reference: male)	0.48 (0.22-1.06)	.07
Hispanic (reference: non-Hispanic White)	1.62 (0.85-3.11)	.15
Non-Hispanic Black (reference: non-Hispanic White)	2.42 (1.23-4.77)	.01
Non-Hispanic Other (reference: non-Hispanic White)	1.99 (0.81-4.87)	.14
GED (reference: High School diploma)	1.12 (0.46-2.72)	.81
College degree (reference: High School diploma)	0.93 (0.34-2.51)	.88
National Guard (reference: Regular Army)	1.58 (0.78-3.18)	.20
Reserve (reference: Regular Army)	0.97 (0.27-3.47)	.97
Lifetime substance use disorder at baseline	2.38 (1.39-4.08)	.002
Combat exposure (0-11)	1.17 (1.04-1.32)	.01
Physical assault since baseline survey	2.17 (0.79-5.96)	.14
Sexual assault since baseline survey	0.48 (0.10-2.20)	.35
Other life-threatening event since baseline survey	1.17 (0.65-2.11)	.61
Exposed to traumas of other people since baseline survey	1.52 (0.87-2.66)	.15
Serious injury or death of loved one since baseline survey	1.61 (0.97-2.67)	.07

© 2023 Campbell-Sills L et al. *JAMA Network Open.*

eReferences

1. Ursano RJ, Colpe LJ, Heeringa SG, et al. The Army Study to Assess Risk and Resilience in Servicemembers (Army STARRS). *Psychiatry*. 2014;77(2):107-119.
2. Stanley IH, Chu C, Gildea SM, et al. Predicting suicide attempts among U.S. Army soldiers after leaving active duty using information available before leaving active duty: Results from the Study to Assess Risk and Resilience in Servicemembers-Longitudinal Study (STARRS-LS). *Mol Psychiatry*. 2022;27(3):1631-1639.
3. Kessler RC, Ustun TB. The World Mental Health (WMH) Survey Initiative Version of the World Health Organization (WHO) Composite International Diagnostic Interview (CIDI). *Int J Methods Psychiatr Res.* 2004;13(2):93-121.
4. Weathers F, Litz B, Herman D, Huska J, Keane T. The PTSD Checklist (PCL): Reliability, Validity, and Diagnostic Utility. International Society for Traumatic Stress Studies; 1993; San Antonio, TX.
5. Blevins CA, Weathers FW, Davis MT, Witte TK, Domino JL. The Posttraumatic Stress Disorder Checklist for DSM-5 (PCL-5): Development and initial psychometric evaluation. *J Trauma Stress.* 2015;28(6):489-498.
6. Kessler RC, Santiago PN, Colpe LJ, et al. Clinical reappraisal of the Composite International Diagnostic Interview Screening Scales (CIDI-SC) in the Army Study to Assess Risk and Resilience in Servicemembers (Army STARRS). *Int J Methods Psychiatr Res.* 2013;22(4):303-321.
7. Posner K, Brown GK, Stanley B, et al. The Columbia-Suicide Severity Rating Scale: Initial validity and internal consistency findings from three multisite studies with adolescents and adults. *Am J Psychiatry*. 2011;168(12):1266-1277.

© 2023 Campbell-Sills L et al. *JAMA Network Open.*
