# Supplementary material for: Exposure to Bullying or Hazing During Deployment and Mental Health Outcomes Among US Army Soldiers
Source: JAMA Netw Open. 2023 Jan 24;6(1):e2252109. doi: 10.1001/jamanetworkopen.2022.52109 (PMC10408263; doi:10.1001/jamanetworkopen.2022.52109)
Supplement: Supplement 2. — Data Sharing Statement [file jamanetwopen-e2252109-s002.pdf]

Data Sharing Statement

Campbell-Sills. Exposure to Bullying or Hazing During Deployment and Mental Health Outcomes Among US Army Soldiers. *JAMA Netw Open*. Published January 24, 2023. doi:10.1001/jamanetworkopen.2022.52109

Data
Data available: No
